# Constitutive metabolomic profile of a transgressive segregant of rice with superior salinity tolerance potentials due to unique morphological features and well-modulated growth

**DOI:** 10.1007/s00425-025-04811-0

**Published:** 2025-08-29

**Authors:** Isaiah Catalino M. Pabuayon, Md. Mamunur Rashid, Ai Kitazumi, Kevin R. Cushman, Habtom W. Ressom, Benildo G. De los Reyes

**Affiliations:** 1 Department of Plant and Soil Science, Davis College of Agricultural Sciences and Natural Resources, Texas Tech University, Lubbock, TX USA; 2 Department of Oncology, Lombardi Comprehensive Cancer Center, Georgetown University Medical Center, Washington, DC USA

**Keywords:** Constitutive salt tolerance, Lipidome, Metabolome, Plant architecture, Resilience vs Response, Rice, Transgressive segregation

## Abstract

**Abstract:**

Understanding the nature of non-parental phenotypes created by transgressive segregation is important in creating novel genetic recombinants that can withstand different environmental conditions for crop production. FL510, a transgressive salinity-tolerant rice genotype from a cross between IR29 (salt-sensitive) and Pokkali (salt-tolerant), has tolerance mechanisms active under control conditions and improves survival upon the onset of salinity. This study compares normal-state metabolomes and lipidomes of FL510 with its parents. Principal component analysis (PCA) of the identified analytes showed clear and expected similarity between FL510 and Pokkali, while partial least squares discriminant analysis (PLS-DA) emphasized overlaps between the metabolic profiles of IR29 and FL510. The analysis identified metabolites with inherited patterns of abundance from either parent in FL510 and those with unique, non-parental abundances, and these were supported by differential expression of key pathway-related genes identified through transcriptome analysis. Strigolactone precursor production was identified as a key feature in FL510, which may help explain its unique architecture that is beneficial for osmotic stress. We also identified a divergence between productivity under ideal environments leading to free radical production versus tempered production that offers better survival under marginal growing conditions. FL510 showed an inheritance of hormone and amino acid abundances from Pokkali, which further explains some of its architectural and previously studied stress-response features. Meanwhile, the similarity of FL510 with IR29 in terms of flavonoid indicates an inheritance of productivity and is consistent with previous reports of induction for these molecules under stress, rather than being active under control conditions.

**Main Conclusion:**

Through repeated genetic recombination of genetically distant alleles, the transgressive segregant FL510 gained unique, non-parental signaling pathways and complementary metabolome features from both parents leading to positive net genetic gains.

**Supplementary Information:**

The online version contains supplementary material available at 10.1007/s00425-025-04811-0.

## Introduction

In plant breeding, superior or inferior individuals that are beyond the phenotypic boundaries of the parents can be created as an outcome of the phenomenon of transgressive segregation. One of the major causes of this phenomenon is the positive impact of novel combinations of genes or alleles in a compatible genetic background (Bradshaw 2017). Transgressive individuals occur in nature as a consequence of natural hybridization between parents with wide genetic divergence, and those rare individuals are believed to fuel adaptive evolution under certain ecological niches. Creation of novel phenotypes allows new genotypes to flourish under environments that the majority of the population would not typically inhabit (Rieseberg et al. 1999; de Los Reyes 2019). It has been proposed that transgressive individuals could serve as a raw material for the creation of a new phylogenetic lineage forming the bridge between the starting population and the new niche to be occupied. In positive transgressive individuals, it has been shown that the combination of parental attributes can either be additive or complementary, resulting in net positive phenotypic gain. In negative transgressive individuals, the cumulative effects of the negative attributes of the parents collectively drag the phenotypic potential, with the detrimental effects greater than the collective impacts of the positive attributes of each parent, hence negative net genetic gain.

In plant breeding, transgressive segregation is a powerful tool for creating non-parental novelties, especially for complex traits that contribute to enhanced resilience to sub-optimal environmental conditions. In our earlier studies, we have shown that the inherent/constitutive growth potential of a genotype and its modulation under stress play an important role in overall stress tolerance potential by virtue of genetic mechanisms that balance the energy demands of growth-related processes with defense-related responses. It has been shown that adaptive morphological and developmental traits, i.e.*,* ideotypes, confer large net gains in stress tolerance potentials under salinity stress conditions (Pabuayon et al. 2020, 2021).

Physiological trade-offs that affect the intricate balance between growth, development, and defense-related responses are an important consideration when identifying and selecting for the most critical traits that should be stacked in a single genotype by backcross breeding. A classic example of such trade-offs is the selection against plant height during the Green Revolution along with other morphological traits such as higher tillering and panicle number, which brought greater impacts to the source-sink balance between vegetative source and reproductive sinks under optimal and ideally managed environments (Khush 1999). Another example is the choice between delayed flowering (avoidance) or early flowering (escape) during stress. In Arabidopsis, for example, flowering under drought is photoperiod-dependent, as shorter days trigger flowering repressors, while long days accelerate flowering (Kazan and Lyons 2016). In rice, prolonged drought stress delays flowering, which eventually exhausts available photosynthate for floral and seed development, severely compromising grain yield. Quantitative trait loci (QTL) that maintain flowering time have positive effects on the maintenance of productivity, albeit at an understandably lower magnitude (Sanchez et al. 2022). Trade-offs are expected due to the finite cellular resources, which become smaller and easily depleted under stress conditions.

In the face of changing environments affecting agricultural productivity, there is a push towards breaking the genetic bottleneck created during the Green Revolution to create a complex balance between growth-related and defense-related responses and maintain vegetative and reproductive productivity under sub-optimal environments (de Los Reyes 2019). The inherent characteristics of a genotype for a well-primed response to stress without significant compromise to growth (i.e., balancing the trade-offs between growth-related and defense-related responses) should be explored and one possible way to achieve this is through the phenotypic outliers or transgressive segregants. We have previously identified a transgressive segregant (FL510) from a recombinant inbred population (F_9_, F_10_) of rice derived from hybridization of the genetically diverse cultivars IR29 (salt-sensitive) and Pokkali (salt-tolerant). The transgressive segregant FL510 has superior tolerance to hyper-salinity by virtue of multiple mechanisms that contribute to maximal ion exclusion capacity conferred by its overall morphological characteristics and inherent capacity for modulated growth (Pabuayon et al. 2020, 2021). Previous analysis of its stress response metabolome at moderate coverage indicated that it has similarities with the sensitive parent IR29, supporting the hypothesis that genetic contributions from both parents are necessary in creating recombinants with superior traits. There remains the question of where the initial metabolome constitution of FL510 lies and how this starting point differs from the parents. We hypothesized that FL510 has a unique natural-state metabolomic composition that contributes to better priming of well-modulated growth that is compatible with defense responses. This unique composition leads to true resilience that increases its survivability when combined with typical stress responses such as salt exclusion, osmolyte production, and free radical scavenging.

In this study, we investigated the genotype-specific constitutive metabolomic signatures of transgressive segregant FL510, which is superior to its parents with respect to various growth characteristics that provide an adaptive advantage under salinity stress. Specifically, our aim was to profile the metabolomic signatures during the most active stage of vegetative growth as a window to understand the inherent genotype-specific and shared profiles between the transgressive segregant and its parents, specifically within the context of the contributions of its inherently well-modulated growth potential to its superior salinity stress tolerance potential. We performed direct comparisons of the natural-state metabolomes and lipidomes of the parents IR29 and Pokkali, and their transgressive progeny FL510. These profiles revealed the unique, non-parental metabolic profiles of the transgressive progeny. In conjunction with transcriptome data, we analyzed the differences between the three genotypes in an integrative manner. Our findings highlight the importance of the contributions of both parents, underscoring the contributions not just from the salinity-tolerant parent Pokkali but also from the growth potential of the salinity-sensitive but vigorously growing parent IR29. Based on metabolomic signatures, we uncovered potential physiological trade-offs between a landrace, Pokkali, and a modern variety, IR29, which are tempered and modulated in the transgressive progeny FL510.

## Materials and methods

### Plant materials and growing conditions

The rice genotypes used in this study were IR29, a salinity-sensitive Green Revolution cultivar, Pokkali, a salinity-tolerant landrace traditionally grown in salt marshes of India, and FL510, a transgressive recombinant inbred line (RIL) that shows an extreme level of tolerance to salinity (Pabuayon et al. 2021). Seed dormancy was broken at 50 °C for 5 days, then rested at room temperature before germinating on a culture media containing ½ strength Murashige-Skoog (MS) nutrient solution and 1% (w/v) sucrose. Germinated seeds were grown for two weeks in the culture media and then transplanted to pots with Turface MVP (calcine clay media), supplemented with hydroponics solution with 1 g L^−1^ Peter’s 20–20-20 and 0.4 g L^−1^ FeSO_4_·7 H_2_O at pH 5.5. Seedlings were grown in a controlled chamber at 28°C/25°C a 12-h day/night cycle, with weekly changes of nutrient solutions. Older leaf blade tissues from two nodes after the emerging meristem (three biological replicates per genotype) were sampled for metabolomic and lipidomic profiling two weeks after transplanting (1 month after germination started). These were to ensure that the leaves were more similar developmentally to each other and exclude younger leaves that grew more recently. Leaf sheaths were not included. Plants for transcriptome analysis were grown in UC soil mix in the greenhouse under the same day and temperature regimes and were sampled at the same developmental stage (1 month after germination started) as the plants used for metabolomic and lipidome analyses. RNA was extracted using the E.Z.N.A.® Plant RNA Kit from Omega Bio-tek (Norcross, GA, USA). RNA-Seq was performed by Genewiz (South Plainfield, NJ, USA) on the Illumina platform with paired-end sequencing at 150bp reads.

### Metabolite extraction

Pulverized leaf tissues (100 mg) were combined with a mixture of ice-cold chloroform:methanol:water (1:2.5:1, by vol.) containing internal standards. Debrisoquine sulfate and 4-nitrobenzoic acid (Sigma-Aldrich) were used as internal standards at a concentration of 2 µg mL^−1^ for metabolomics profiling in both positive and negative modes, respectively, while PC(16:0/18:1)-d31 (Avanti Polar Lipids; Alabaster, AL, USA) and arachidonic acid-d8 (Sigma-Aldrich) were used as internal standards at concentrations of 1 µg mL^−1^ for lipidome analysis in positive and negative modes, respectively. Sample mixtures were homogenized with vortexing and sonication. Aqueous and organic phases were separated through centrifugation at 22,000 *g* at 4 °C for 10 min.

For metabolome profiling, the aqueous layer was reconstituted with 100 µL 50% (by vol.) methanol in water with 0.1% (by vol.) formic acid. For lipidome profiling, the organic layer was reconstituted with 100 µL isopropanol:acetonitrile:water (2:1:1, by vol.). An aliquot of 5 µL of the reconstituted solution was injected into the UHPLC-Q-Exactive-MS system. Quality control samples for evaluating equipment stability were created by combining equal volumes of reconstituted metabolome and lipidome samples.

### Instrument settings and conditions for metabolome profiling

The Vanquish UHPLC system connected to a Q-Exactive mass spectrometer (Thermo Fisher Scientific) equipped with a heated electrospray ionization (HESI) source was used for all profiling runs in both positive and negative modes as previously described in Rashid et al. (2023), with a few modifications. For metabolome profiling, chromatographic separation made use of the ACQUITY UPLC BEH C18 column (2.1 × 100 mm, 1.7 mm; Waters, Milford, MA, USA). The autosampler and column oven temperature were maintained at 4 °C and 40 °C, respectively. The mobile phase was comprised of a mixture of 0.1% formic acid in water (by vol.; mobile phase A) and in methanol (by vol.; mobile phase B), with elution at a flow rate of 0.3 mL min^−1^ in an 18-min gradient. The gradient started with 5% B for 0.5 min and gradually increased to 20% B at 3 min, 70% B at 5 min, and 100% B at 13 min. The elution with 100% B was maintained until 15.5 min and then rapidly reduced to the initial gradient (5% B) at 16.0 min for a 2 min equilibration phase. The volume of the injection was adjusted to 5 µL. For the lipidome profiling, chromatographic separation was performed with an ACE Excel 2 Super C18 column, 2.1 × 100 mm, 1.7 mm (Advanced Chromatography Technologies Ltd., Aberdeen, Scotland, UK). The elution solvent consisted of 10 mM ammonium acetate, either in 40% acetonitrile (by vol., mobile phase A) or a mixture of acetonitrile and isopropanol (1:9, by vol., mobile phase B). The elution gradient mix began with a 40% solvent B for 1 min, then rapidly escalated to 65% solvent B at 3.5 min, followed by a steady increase to 100% solvent B at 13 min. It remained at 100% solvent B for 0.5 min before rapidly returning to the initial gradient at 14 min. The system was brought back to its starting gradient and allowed to stabilize for 2 min. The autosampler and column oven temperature were maintained at 4 °C and 50 °C, respectively.

For both metabolome and lipidome profiling, leaf samples were analyzed using a 66.6–1000 *m/z* and 80–1200 *m/z* scan range, respectively. Full MS data was acquired at a resolution of 70,000 with a centroid mode, while the MS/MS data was acquired at a resolution of 17,500 using 10 loop counts by applying stepped normalized collision energy (step-NCE) of 20, 30, and 45 with an isolation window of 2.0 *m/z*. Dynamic exclusion was set to 30.0 s, and the automatic gain control (AGC) was set to 1e^6^ and 1e^5^ for full MS and dd-MS2, respectively. Parameters for the heated electrospray ionization (HESI) source were as follows: capillary temperature at 320 °C, spray voltage at 3.8 kV for positive ion mode and 3.2 kV for negative ion mode, sheath gas flow rate at 45.0 arb and 35.0 arb for positive and negative ion modes, respectively, auxiliary gas flow rate at 10.0 arb for positive ion mode and 1.0 arb for negative ion mode, and sweep gas flow rate at 1.0 arb for positive ion mode and 0.0 arb for negative ion mode. Nitrogen was employed both as sheath and auxiliary gases. Xcalibur 4.4 and Freestyle 1.7 software packages were used to analyze the mass spectral data.

### Metabolome data processing and analysis

The raw spectral data (i.e., chromatographic peak detection, MS spectrum detection, deconvolution of overlapping ions, and integration of peak areas) was analyzed with Compound Discoverer 3.3 software (Thermo Fisher Scientific) was used to analyze the raw data and for the identification of putative metabolites by applying several filters, including background subtraction, MS^*n*^ fragmentation information, ΔMass (± 5 ppm). Putative identities of metabolites were determined by matching the data with several databases, including mzCloud (https://www.mzcloud.org/), Chemspider (Pence and Williams 2010), Metabolika, and other information resources in the public domain. Other tools and spectral libraries, including MetaboQuest (https://tools.omicscraft.com/aiSysMet/), LipidSearch (Thermo Fisher Scientific), Human Metabolome Database (Wishart et al. 2022), and METLIN (Guijas et al. 2018), were also used to identify putative metabolites based on the *m/z* of the mass adducts ([M + H]^+^, [M + Na]^+^, [M + NH^4^]^+^, [M − H]^−^, etc.) and MS/MS fragments of each *m/z*. The internal standards added during sample preparation were used to normalize positive and negative lipidomics data. Principal component analysis (PCA) and partial least square discriminant analysis (PLS-DA) were performed with Metaboanalyst 6.0 (Pang et al. 2024), following log transformation and Pareto scaling to visualize and assess the differential metabolites among groups. ANOVA and Tukey’s post-hoc HSD test were used to determine metabolites with significant differences among the genotypes (*P* < 0.05). Statistical analysis and plot visualization were done using R software v4.4.0 (R Core Team 2024).

### Transcriptome analysis by RNA-seq

RNA-seq data were processed using a novel pipeline for inter-genotypic comparison. Adapter and low-quality bases at Q30 were first trimmed from raw reads with sickle v1.33 with a minimum length of 100-bp (Joshi and Fass 2011). Preprocessed reads were subjected to reference-guided de novo assembly against the IR8 (GCA_001889745.1) and N22 (GCA_001952365.3) genomes by Trinity v2.13 (Haas et al. 2013; Stein et al. 2018). Candidate open reading frames from de novo assembled transcripts were predicted with TransDecoder v5.5. Predicted protein sequences were anchored back to the genomes using MetaEuk v5-34c21f2 (Levy Karin et al. 2020). Annotation of the reference genomes was also conducted with MetaEuk using reference protein sequences (IRGSP-1.0_2024-07–12; Sakai et al. 2013).

To equalize the gene length across genotypes, the anchored read regions that were commonly detected across all genotypes (overlapping between IR29, Pokkali, and FL510) were adopted as conserved transcribed regions using bedtools v2.30 (Quinlan and Hall 2010). Gene models developed from overlapped features were used for counting reads using featurecount from reference-based mapping generated by HISAT2 v2.1.0 with option ‘-k 1’ (Liao et al. 2014; Kim et al. 2015). Gene expression was compared across genotypes using EdgeR v3.28.1 (Robinson et al. 2010). Comparison was based on the gene model of a conserved region established from overlaps at a cutoff value (FDR < 0.05). Compound and pathway IDs were assigned using the KEGGREST package v1.46 (Tenenbaum et al. 2019).

## Results

### Contributions of parental metabolomes and lipidomes to the transgressive nature of FL510

**Fig. 1:**
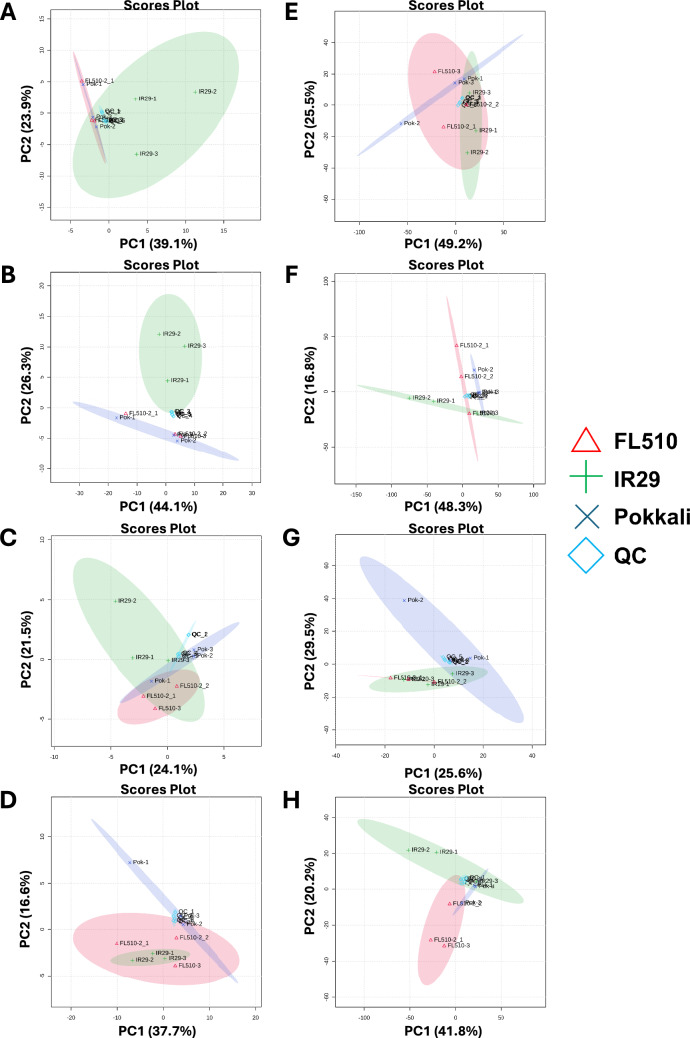
Principal component analysis and partial least squares discriminant analysis of legethe metabolome and lipidome datasets. Sample clustering analysis was done using principal component analysis (PCA; **A**, **B**, **E**, and **F**) and partial least squares discriminant analysis (PLS-DA; **C**, **D**, **G**, and **H**) for the positive (**A**, **C**, **E**, and **G**) and negative (**B**, **D**, **F**, and **H**) ion modes of the metabolome and lipidome datasets to determine consistency and similarity between the samples used in the analysis. **A**, **C** Positive metabolome, *n* = 6303. **B**, **D** Negative metabolome, *n* = 4759. **E**, **G** Positive lipidome, *n* = 3963. **F**, **H** Negative lipidome, *n* = 2576. The analysis also included five samples comprising of a combination of samples from the 3 genotypes tested as quality control. The different genotypes are indicated by different symbols for each (IR29, green cross; Pokkali, blue cross; FL510, red triangle; QC, blue diamond). Colored ovals represent confidence intervals (*P* < 0.05) for the clusters observed between the samples

QC samples from the comparative panel (IR29, Pokkali, FL510) were distributed between the three genotypes in terms of chromatogram peaks and in the principal component (PC) plots of metabolite abundances (Fig. 1; Suppl. Fig. S1). Trends were consistent in both the positive and negative ion modes of the metabolome profiles and in the positive and negative ion modes of the lipidome profiles. A total of 9,892 and 7,459 analytes, respectively, were detected in the positive and negative ion modes of the metabolome profiles. A total of 5,688 and 4,235 analytes, respectively, were detected in the positive and negative ion modes of the lipidome profiles. After filtering low confidence data points, the total number of detected analytes were reduced to 6,303 (positive ion mode) and 4,759 (negative ion mode) in the metabolome profiles. Filtration of low-confidence data points in the lipidome profiles led to a total of 3,963 and 2,576 analytes in the positive and negative ion modes, respectively.

PCA and PLS-DA revealed the extent of similarity in the metabolome and lipidome composition across genotypes. Unsupervised clustering across genotypes by PCA revealed the strongest trends in terms of metabolite abundance. PLS-DA (“supervised” PCA) takes advantage of well-defined classes among the genotypes to emphasize the differences across, in terms of metabolite abundances. PCA plots of the positive ion mode of the metabolome datasets revealed strong similarities between the superior parent Pokkali and the transgressive progeny FL510, while the inferior parent IR29 exhibited much lower similarity to both Pokkali and FL510 (Fig. 1A). The PCA of the negative ion metabolome showed similar trends, but with IR29 having a clearer separation from Pokkali and FL510 (Fig. 1B). Interestingly, FL510 has a greater overlap with IR29 than with Pokkali in the PLS-DA plots (Fig. 1C and D). This trend is especially true for the negative ion mode metabolites, indicating a strong similarity in metabolite abundances between the inferior parent IR29 and the transgressive progeny FL510. These trends revealed that the superior nature of the transgressive FL510 is not without the contributions from its inferior parent IR29.

PCA of the positive ion mode of the lipidome profiles indicated a picture of more consistent parental contributions to the overall features of FL510, with the inferior parent IR29 and superior parent Pokkali covering distinct areas that are encompassed wholly by the plots of FL510 (Fig. 1E). In contrast, the negative ion mode of the lipidome profiles indicate a much clearer distinction and minimal overlap across all three genotypes (Fig. 1F). Thus, the negative ion mode of the lipidome profile tends to represent a clear picture of the novel transgressive signatures in the progeny FL510 that is not in both parents. The PLS-DA plot of the positive ion mode of the lipidome profiles also emphasized the overlap between IR29 and FL510 and the differences between FL510 and Pokkali (Fig. 1G). The PLS-DA plot of the negative ion mode of the lipidome profiles also supported the corresponding PCA plot in the distinction of FL510 from parents (Fig. 1H). However, there are similarities between the two parents that are also visible in the PCA plots. Overall, the data support a clear distinction of the metabolome and lipidome profiles of FL510 based on the specific parental features that were combined.

A total of 2,464 analytes with statistically significant differences between the genotypes were identified, of which 815 and 721 were from the positive and negative ion modes, respectively, of the metabolome profiles, and a total of 608 and 320, respectively, from the positive and negative ion modes of the lipidome profiles (Suppl. Table S1). There was a strong similarity in the abundance patterns between Pokkali and FL510, which reflects the overlaps observed in the PCA plots between them (Suppl. Fig. SS). This also implies that the similarities between IR29 and FL510 are from background metabolites that are not significantly different in terms of abundance. More similarities between Pokkali and FL510 support findings that show a higher inheritance of genetic features from Pokkali to FL510 (Kitazumi et al. 2024).

### Novel profile of a strigolactone signaling pathway in the transgressive FL510

**Fig. 2:**
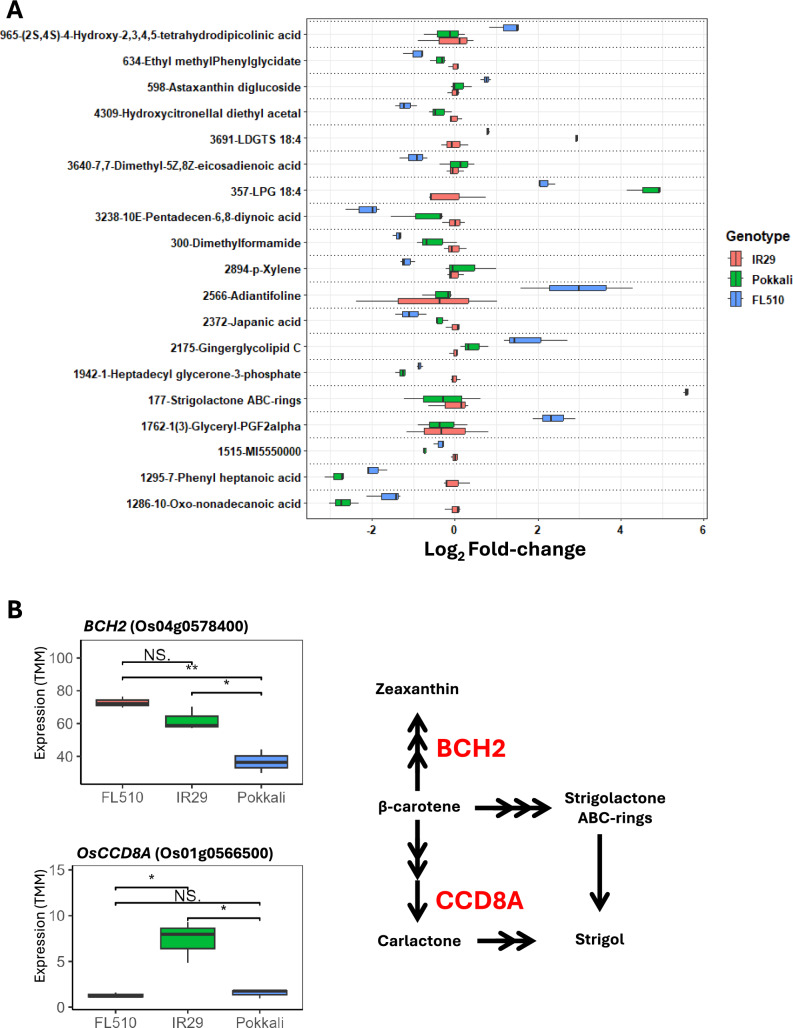
Annotated metabolites that display unique induction or repression in FL510 compared to IR29 and Pokkali and the expression of key genes potentially related to strigolactone biosynthesis. (**A**) Metabolites that showed significant difference in abundance in FL510 compared to Pokkali and IR29 are shown. These metabolites have *P*-values less than 0.05 in the comparisons between FL510 and Pokkali and FL510 and IR29 and thus have non-parental abundance patterns. Abundance values are shown as log_2_-fold change ratios with respect to IR29 values (i.e., log_2_(genotype/IR29). Unannotated metabolites are excluded from this figure. Majority of the metabolites shown have similar abundance patterns between IR29 and Pokkali, indicating potential conserved characteristics that are changed in the transgressive FL510. (**B**) The expression (TMM) of *BCH2* (Os04g0578400) and *OsCCD8A* (Os01g0566500). These are components of the carotenoid biosynthesis pathway close to the synthesis of strigolactones, as shown in the accompanying reaction diagram. Pairwise comparisons for expression are also shown. Asterisks (*) denote significant differences (**, *P* < 0.01; *, *P* < 0.05)

We sought to identify metabolites with unique, non-parental abundance in FL510 that may contribute to its unique plant architecture. There were 50 metabolites with such abundance patterns (Suppl. Table S1). Of these, 19 have been annotated in the metabolite databases (Fig. 2A). Strigolactone ABC-rings, which form the backbone of strigolactone hormones, were highly abundant in FL510 compared to the parents (~ sixfold; Fig. 2A). High levels of strigolactone precursor may indicate that FL510 features a novel and unique signaling pathway for growth and development that were not directly attributed to parental features. Mutants for strigolactone biosynthesis have been shown to display altered tillering and overall architecture, which can also be observed in FL510, especially with respect to its parents.

Two genes related to the strigolactone metabolic pathway were differentially expressed in FL510 relative to its parents IR29 and Pokkali (Fig. 2B). A β-carotene hydroxylase gene (*BCH2*; Os04g0578400) is downregulated in Pokkali compared to FL510. This gene is involved in the conversion of β-carotene to other carotenoids such as zeaxanthin. Higher expression of this gene may reduce the creation of strigolactone-ABC rings, an opposing multistep conversion process from β-carotene. Meanwhile, *OsCCD8A* (carotenoid cleavage dioxygenase 8 A; Os01g0566500) is upregulated in IR29 compared to both Pokkali and FL510. This enzyme is part of the multi-step process in converting β-carotene to carlactone, a type of strigolactone precursor (Seto et al. 2014).

### Metabolome contributions of IR29 to FL510 indicate potential damage markers from increased productivity

**Fig. 3:**
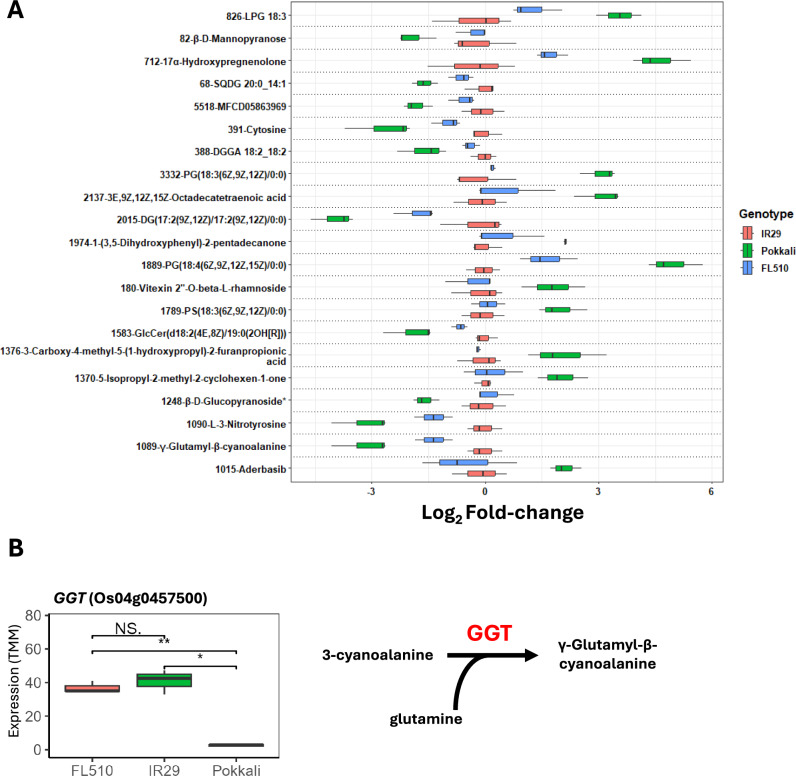
Annotated metabolites that have shared abundance patterns between FL510 and IR29 and the expression of a key enzyme for cyanoamino acid metabolism (GGT) across the three genotypes. (**A**) Metabolites with significant difference in abundance in IR29 and FL510 compared to Pokkali are shown. Abundance values are shown as log_2_-fold change ratios with respect to IR29 values (i.e., log_2_(genotype/IR29). These metabolites have *P*-values less than 0.05 in the comparison between FL510 and Pokkali, and IR29 and Pokkali, while having *P*-values more than 0.05 in the comparison between FL510 and IR29. Unannotated metabolites have been excluded from this figure. The asterisk (*) for β-D-glucopyranoside denotes an abbreviation for its full name, which is 2-[(2R,4aS,6S,8aS)−6-hydroxy-4a-methyl-8-methylenedecahydro-2-naphthalenyl]−2-propanyl beta-D-glucopyranoside. (**B**) Expression values (TMM) of *GGT* (Os04g0457500) in FL510, IR29, and Pokkali alongside the specific reaction it catalyzes. Pairwise comparisons for expression are also shown. Asterisks (*) denote significant differences (**, *P* < 0.01; *, *P* < 0.05)

There is a significant proportion of metabolites in the negative ion mode of the lipidome profiles with comparable abundances between IR29 and FL510, and these similarities appear to drive the distinctions shown in Fig. 1G. There were 70 metabolites that were identified with similar abundances in IR29 and FL510, but significantly different from Pokkali. These metabolites potentially represent gains through the features inherited from IR29. Based on the annotation, these metabolites include glycerolipids such as phosphatidylserine PS(18:3(6Z,9Z,12Z)/0:0), phosphatidylglycerol PG(18:4(6Z,9Z,12Z,15Z)/0:0), PG(18:3(6Z,9Z,12Z)/0:0), diradylglycerol DG(17:2(9Z,12Z)/17:2(9Z,12Z)/0:0), which were clearly more abundant in superior parent Pokkali compared to the transgressive progeny FL510 and inferior parent IR29 (Fig. 3A). The precise functions of these lipid metabolites in plant growth and defenses are currently unknown. Few studies have shown that differential glycosylation and biosynthesis of these lipids can influence chloroplast function, as they are components of the thylakoid membranes (Kobayashi 2016). While four genes related to fatty acid metabolism showed significant expression differences among the genotypes (Os11g0659500; Os07g0620500; Os02g0503500; Os08g0327400/*OsENR1*), there was no clear pattern of inter-genotypic expression differences relating to the metabolites identified (Suppl. Fig. S3). The L-3-nitrotyrosine showed inter-genotypic differences in abundances, being significantly at lower level in Pokkali (~ threefold decrease) compared to FL510 and IR29 (~ 1.5-fold decrease, non-significant). γ-Glutamyl-β-cyanoalanine is another metabolite with similar inter-genotypic similarities as L-3-nitrotyrosine. This metabolite has been implicated as a product of cyanide catabolism, which can arise from the production of ethylene, an important plant growth hormone (Seo et al. 2011; O’Leary et al. 2014). Interestingly, the transcriptome data also indicated that a gene encoding γ-glutamyl-transpeptidase (*GGT;* Os04g0457500) was downregulated in Pokkali relative to IR29 and FL510 (Fig. 3B). The GGT enzyme combines glutamate and β-cyanoalanine as part of glutathione cycling (Masi et al. 2015).

Some flavonoids had significantly lower abundances in transgressive progeny FL510 and inferior parent IR29 relative to Pokkali. For example, carvotanacetone (5-isopropyl-2-methyl-2-cyclohexen-1-one) and a resorcinol (1-(3,5-dihydroxyphenyl)−2-pentadecanone) were significantly more abundant in Pokkali (Fig. 3A). Vitexin 2″-O-rhamnoside 7-O-methyltransferase ((1S)−1,5-anhydro-2-O-(6-deoxy-α-L-mannopyranosyl)−1-[5,7-dihydroxy-2-(4-hydroxyphenyl)−4-oxo-4H-chromen-6-yl]-D-glucitol), a flavonoid, is also significantly higher in Pokkali. Under their natural states, FL510 and IR29 have less flavonoids, but stress induces their levels. In contrast, Pokkali has higher baseline flavonoid content to begin with. This may impede the production of such molecules under stress, likely due to feedback inhibition, especially under resource-limited conditions. Additionally, the lower flavonoid content in IR29 and FL510 may fit with the *GGT* expression, as antioxidants are consumed more in IR29 and FL510 compared to Pokkali.

### Inherited metabolome features from Pokkali point to divergences between landraces and modern varieties

**Fig. 4:**
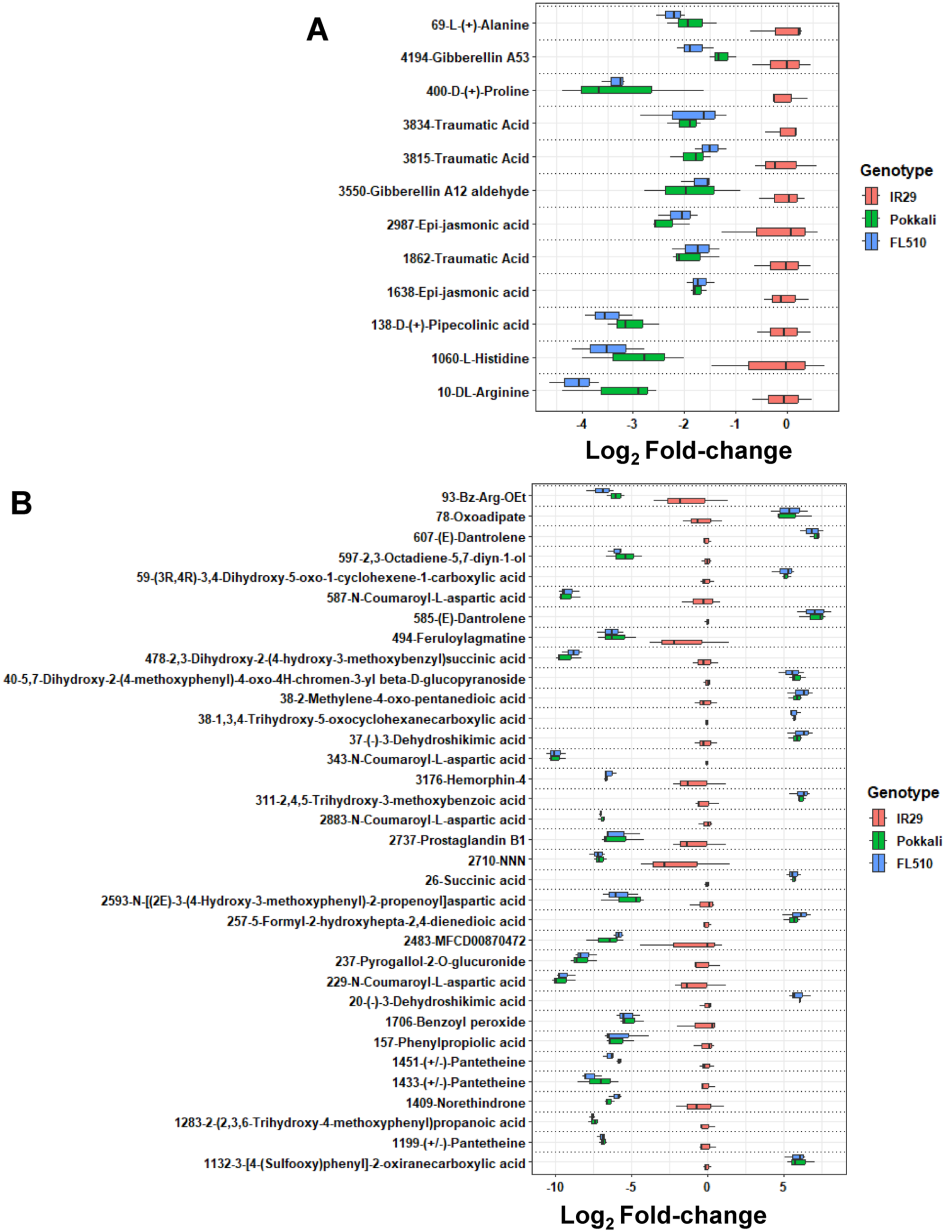
Annotated metabolites that have shared abundance patterns between FL510 and Pokkali. Metabolites with significant difference in abundance in FL510 and Pokkali compared to IR29 are shown. These metabolites have *P*-values less than 0.05 in the comparison between FL510 and IR29, and IR29 and Pokkali, while having *P*-values more than 0.05 in the comparison between FL510 and Pokkali. Abundance values are shown as log_2_-fold change ratios with respect to IR29 values (i.e., log_2_(genotype/IR29). (**A**) Selected amino acids and hormones that have established roles for stress tolerance among the metabolites with significant abundance differences in FL510 and Pokkali compared to IR29. These include alanine, proline, histidine, and arginine, which can serve as osmolytes or as important conduits for osmolyte production. Hormones include traumatic acid (TA), jasmonic acid (JA), and gibberellins. (**B**) Metabolites that have a −5 or 5 log_2_ fold-change in Pokkali and FL510 compared to IR29 are shown. Unannotated metabolites are not shown

**Fig. 5:**
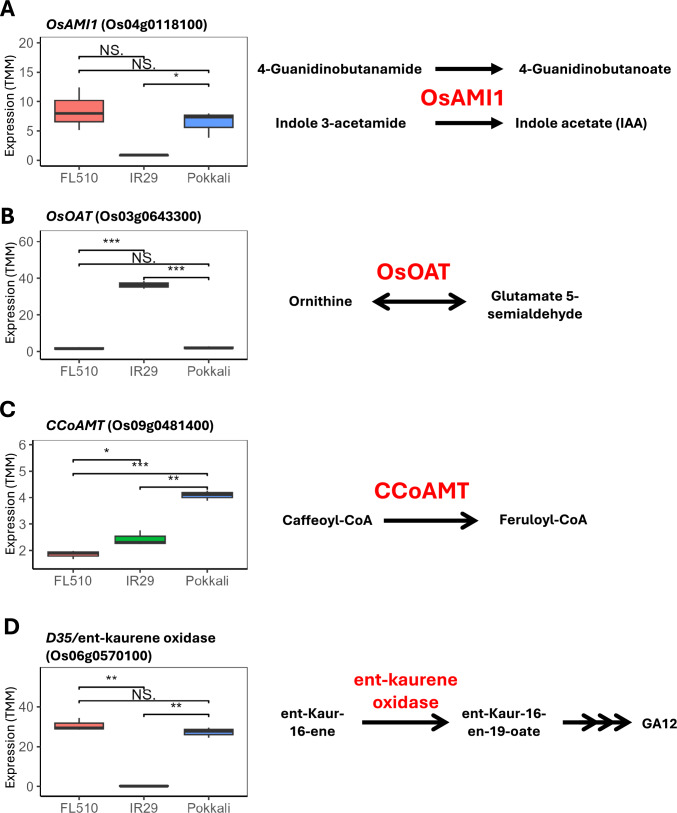
Expression of key genes related to the synthesis of selected metabolites shown in Fig. 4. The expression (TMM) of genes in the biosynthesis pathways of important metabolites highlighted from Fig. 4 are shown, alongside the specific reaction that they catalyze. (**A**) The expression for *OsAMI1* (Os04g0118100), which is involved in arginine/proline metabolism and auxin synthesis in the tryptophan metabolism pathway. (**B**) The expression of *OsOAT* (Os03g0643300), which is also part of the arginine/proline metabolism pathway for nitrogen cycling. (**C**) The expression of *CCoAMT* (Os09g0481400), which is part of the flavonoid biosynthesis pathway. (**D**) The expression of an *ent-*kaurene oxidase gene, *D35*, which is part of the diterpenoid and gibberellic acid biosynthesis pathway. Pairwise comparisons for expression are also shown. Asterisks (*) denote significant differences (**, *P* < 0.01; *, *P* < 0.05)

Almost half of the differentially abundant metabolites appeared to be directly inherited by FL510 from the superior parent Pokkali (1274; Suppl. Table S1). This is consistent with our earlier findings that the Pokkali genome rather than IR29 has larger contributions to the genomic composition of FL510 (Kitazumi et al. 2024). Several amino acids have lower abundances in FL510 and Pokkali compared to IR29, and these include alanine, proline, histidine, and arginine (Fig. 4A). Non-proteogenic amino acids, amino acid derivatives, and tripeptides, are also included in this group as metabolites with lower abundance in FL510 and Pokkali (Fig. 4B). Arginine was especially downregulated, especially in FL510. Two genes related to arginine metabolism were found to have a unique expression pattern in IR29 (Fig. 5A and B). A gene encoding amidase (Os04g0118100) was downregulated in IR29 compared to Pokkali and FL510, and this gene has been shown to function in converting arginine to 4-guanidinobutanoate. Ornithine δ-aminotransferase (*OsOAT*; Os03g0643300) is upregulated in the inferior parent IR29 relative to the transgressive progeny Pokkali and superior parent FL510, and this gene has an important role in nitrogen remobilization (Liu et al. 2018). Interestingly, 3-dehydroshikimic acid is greatly increased in Pokkali and FL510, which is a point of intersection for many biosynthetic pathways, including aromatic amino acids and secondary metabolites (Fig. 4B). Its increased abundance may coincide with the increase in the other metabolites detected in this set. A gene related to flavonoid biosynthesis, caffeoyl-CoA O-methyltransferase (*CCoAMT*; Os09g0481400) is upregulated in Pokkali (Fig. 5C).

Gibberellins (GA; A12-aldehyde and A53) and jasmonic acid (JA; epi-jasmonic acid) were of significantly lower abundances in the transgressive progeny FL510 and superior parent Pokkali compared to the inferior parent IR29 (Fig. 4A). In particular, the GAs detected in the profiles of the three genotypes are precursors for the biosynthesis of bioactive forms of GA (Gianfagna et al. 1983; Großelindemann et al. 1992). Transcriptome data also indicated that *D35*/ent-kaurene oxidase (Os06g0570100) is downregulated in IR29 relative to FL510 and Pokkali (Fig. 5D). This trend shows another potential shunt in the GA biosynthesis process in the semidwarf genotype, as this gene is responsible for the synthesis of the GA precursors (Itoh et al. 2004). The taller stature of FL510 and Pokkali relative to IR29 is associated with the active consumption of these GA precursors, as opposed to IR29 which has a shunt in their conversion to a bioactive form.

While JA has been observed to be higher in IR29 (Fig. 4A), the overall expression of *TIFY* genes was not significantly different among the three genotypes. Only *GATA19* (Os03g0734900) and *GATA20* (Os06g0698900) were upregulated in FL510 and Pokkali, respectively (Suppl. Fig. S4). JA is also heavily involved in biotic stress responses, and its abundances in the three genotypes may also be related to the repression of pipecolic/pipecolinic acid (threefold) in FL510 and Pokkali (Fig. 4A; Suppl. Table S1). Pipecolic acid is important in mediating systemic immunity by regulating free radicals, such as nitric oxide and ROS (Wang et al. 2018). Like other findings in this study, higher pipecolic acid in IR29 may be indicative of higher free radical content from its stronger metabolic activity under control conditions. Traumatic acid, typically characterized as a wounding hormone, is also less abundant in FL510 and Pokkali (Fig. 4A).

## Discussion

The parental genotypes IR29 and Pokkali have enough genetic distance to form a diverse set of progenies, which includes the transgressive line FL510. Previous studies have shown the extreme tolerance of FL510 through the synergism of its morphology and stress response (Pabuayon et al. 2020, 2021). In this study, we investigated the metabolic composition of FL510 in comparison to the parental lines to investigate inherited similarities, differences, and unique patterns in the transgressive line under their natural, unstressed states. Working on the hypothesis that the phenotype FL510 exhibits results from the complementation of positive traits from both parents, we sought to discover what these complementary traits are from a chemical and metabolic composition standpoint.

We identified a novel strigolactone signaling system in FL510, which helps explain its unique architectural features. A key contributor to the unique phenotype of FL510 is the difference in its architecture (i.e., erect tiller angle, wider leaves, and more compact structure) from both parents IR29 and Pokkali (Pabuayon et al. 2020). Its low tiller angle and intermediate growth habit help in reducing osmotic stress, which helps improve survival even under extreme salinity stress at EC = 16 dS m^−1^/~ 160 mM NaCl (Kitazumi et al. 2024). Recent studies have shown that strigolactones can modulate plant architecture in both roots and shoots, and some mutants for strigolactone biosynthesis display altered tillering (Kun et al. 2023; Li et al. 2024). Previous studies showed that the application of synthetic strigolactone (GR24) reduced malondialdehyde and ROS content, which has also been observed in FL510 (Ling et al. 2020; Pabuayon et al. 2021). The results imply that the transgressive progeny FL510 gained a novel strigolactone synthesis and signaling network that may have been configured through repeated recombination events. Given its known physiological roles, it appears that strigolactone could contribute to the unique adaptive morphology and architecture of FL510 and augment other tolerance mechanisms, including ion exclusion and ROS scavenging. The novelty of this signaling pathway in FL510 may also extend beyond the well-explained functions of previously studied strigolactones, considering that there are currently 35 known natural strigolactones with diverse structures and functions (Wang and Bouwmeester 2018; Al-Babili 2024). The expression analysis of genes related to strigolactone synthesis, *BCH2* and *OsCCD8A,* also indicates that biosynthesis shunts from allelic differences inherited from both parents contribute to increased strigolactone precursor production. This is a clear example of novel gene networks created from the genetic recombination of two genotypes with sufficient genetic distance from each other, such as a landrace and a modern variety. Increased *BCH* gene expression helps improve stress tolerance through abscisic acid (ABA) synthesis and photoprotection through xanthophyll cycling (Davison et al. 2002; Du et al. 2010). This may also be a unique response that is quickly activated upon stress induction due to innate differences in expression under control conditions. Meanwhile, the expression of *OsCCD8A* homologs such as *CCD7* and *8* were identified to be involved in strigolactone production and bud development (Rubio-Moraga et al. 2014). Mutagenesis of homologous *CCD* genes in sorghum has also led to reductions in strigolactone production, altering plant architecture (Hao et al. 2023). Taken together, the results suggest that the accumulation of strigolactone-ABC rings in FL510 is because of shifts towards the biosynthesis of other molecules in the carotenoid pathway. Additionally, previous studies have shown that upregulation of *CCD* genes improves stress tolerance (Yi et al. 2023), and higher strigolactone precursor concentrations may help in creating a more efficient response upon the introduction of stress.

This genetic distance is also reflected in the metabolome profiles of the two parents. Global comparisons delineate the two genotypes, especially in terms of gross metabolite content (Suppl. Fig. S2). Flavonoid contents in Pokkali and IR29 differ greatly which is connected to the difference in productivity between the two genotypes (Fig. 2). IR29, a Green Revolution variety, is bred for high photosynthesis and productivity, as can be seen in the higher free amino acid content and the expression of nitrogen cycling genes such as *OsOAT* (Figs. 4A and 5B). This gene functions in the urea cycle, the cycling of nitrogen, and interconversion of amino acids (Witte 2011). Its higher expression may explain the higher concentration of amino acids. Green Revolution varieties are exceptionally responsive to increased fertilizer application, which coincided with the advances in agronomic management techniques. However, higher productivity translates to stronger photosynthesis, which steadily produces free radicals, potentially indicated by the high concentration of 3-nitrotyrosine in IR29 and FL510 (Pospíšil 2009; Foyer 2018). Tyrosine nitrosylation has been suggested as a potential marker of excessive build-up of reactive nitrogen species (RNS) and cellular injuries (Bartesaghi and Radi 2018; Staszek and Gniazdowska 2020). The profile of L-3-nitrotyrosine appears to be inherited by FL510, indicating its high photosynthetic capacity as well. The higher flavonoid concentration in Pokkali also supports this, as flavonoids are antioxidants for free radicals produced in processes such as photosynthesis (Agati et al. 2020). The identified carvotanacetone and resorcinol have also been implicated for antioxidant functions, though their exact function in plant metabolism is currently unknown (Olszowy 2019; Tahri et al. 2022). Similarly, the identified vitexin analog is also a flavonoid known to reduce oxidative damage based on pharmacological studies (Borghi et al. 2013; Lee et al. 2015; Babaei et al. 2020). Vitexin and its analogs have been shown to induce the production of other flavonoids (Guo et al. 2024). The flavonoid content of Pokkali can also contribute to its lower photosynthetic activity by reducing photosynthetic activity through impediment of the Hill reaction (Morales-Flores et al. 2015). *CCoAMT* expression also aligns with the increased flavonoid concentration in Pokkali, as its homolog has been implicated in increased flavonoid expression in citrus (Liao et al. 2023). Additionally, a homolog in rice (OsCOMT1) has been reported to reduce lignin content when silenced (Koshiba et al. 2013). Meanwhile, the high abundance of 3-dehydroshikimic acid in Pokkali and FL510 is also interesting as it is a target of manipulating cell wall lignin contents, which means it may also contribute to the erect plant stature of FL510 and Pokkali (Eudes et al. 2015; Umana et al. 2022).

Free radicals are an important component of the hypersensitive responses against infections. Green Revolution rice can be expected to carry higher free radical contents, as they are bred to be tolerant to biotic stresses, which are primary drivers of the loss of yield potential in well-managed and resource-abundant crop systems (Ladha et al. 2021). The higher pipecolic acid and JA concentration in IR29 supports the biotic stress tolerance of IR29 (Halim et al. 2006; Wang et al. 2018). This represents a trade-off made during the breeding of highly productive modern genotypes, as free radical production is also a major detriment in abiotic stress tolerance. While it is a natural byproduct of metabolic activity and an important component against plant disease, it can also destroy cellular components and lead to cell death (Gechev et al. 2006). Under salinity, ROS and free radical production increase, and the modern variety IR29 cannot sustain its growth. The landrace Pokkali can, because it has built-in shunts against over-productivity and ROS production (i.e. flavonoids). This comes with the cost of lower yield and yield quality, higher vegetative tissue production, lower responsiveness to fertilization, and other traits that were selected against for the Green Revolution. Expression of vegetative growth-related genes and metabolites is also highly contrasting between the two. GA is being utilized in the larger Pokkali and FL510 and auxin biosynthesis also seems stronger with FL510 and Pokkali (Figs. 4A and 5A). Reduction in GA is the primary driver that created the semidwarf rice ideotype as smaller plant height arose from a defective GA-20 oxidase gene (Spielmeyer et al. 2002). The semidwarf rice also had higher GA precursor accumulation (GA53) in the stems, which aligns with the findings here. Traumatic acid may also be contributory to the differences in morphology between the two parents, as it has been shown to interact with auxin, eliciting auxin catabolism and inhibiting root growth in lentil plants (Pilet 1965). It has also been observed to help induce bud initiation in Torenia independent of cytokinin activity (Tanimoto and Harada 1984). Traumatic acid has been studied for its beneficial effects in mitigating salt stress in algae and may have similar effects for plants, potentially through ROS mitigation, which may be important for IR29 because of its increased photosynthetic capacity (Pietryczuk et al. 2014). Additionally, the amidase gene (Os04g0118100) downregulated in IR29 compared to Pokkali and FL510 may also contribute to the unique architecture of FL510 and in the weaker stress response in IR29. This gene is responsible for the conversion of indole-3-acetamide to indole acetate/IAA and potentially relevant to the architectural attribute of FL510 characterized by its uniquely smaller tiller angle (Du et al. 2013; Pabuayon et al. 2020; Li et al. 2021). Meanwhile, 4-guanidinobutanoate was observed to be induced in watermelon by a variety of stresses and has been proposed as a potential tolerance marker due to its abundance in flood-tolerant triploid watermelon (He et al. 2023).

On the global scale, FL510 inherited a vast majority of the metabolite abundance patterns in Pokkali (Suppl. Fig. S2). However, some important metabolites are inherited from IR29 to FL510 in a tempered manner. For example, potential indicators of relative free radical abundance in IR29 mentioned above are generally around onefold lower in FL510 (Fig. 3A), indicating that while there might be similarity in metabolic activity between FL510 and IR29, there is lower severity in FL510. FL510 shows a complementary phenotype when combined with improved ion exclusion inherited from Pokkali. Although there are strong similarities between the metabolomes of FL510 and Pokkali, there are also indicators that there is divergence between them in terms of the gene modules these metabolites act on. For example, *GATA19* is only highly expressed in FL510 despite similarities in JA content between FL510 and Pokkali (Suppl. Fig. S4). Overexpression of *GATA19* has been shown to increase salinity tolerance in transgenic rice, and it may also contribute to FL510’s superior phenotype (Li et al. 2025). JA has also been shown to govern a variety of processes, including ethylene signaling (Nazir et al. 2023; Davison et al. 2022). This may be connected to the observations regarding the higher abundance of γ-glutamyl-β-cyanoalanine in IR29. This may mean that while metabolites appear to be highly skewed for similarity between Pokkali and FL510, the actual gene activities occurring may be very distinct between them.

The study further supports previous findings that both parental lines are contributory towards transgressive phenotypic behavior. The results here also accentuate the multi-faceted nature of stress tolerance, as there is no single gene, metabolite, or network that is solely responsible for transgressive tolerance. Thus, future studies to confirm transgressive behavior will require a combinatorial approach that adds different components identified in this study and in previous works. It also showcases the potential of genetic recombination to create novel phenotypes through new gene interactions. In the context of crop improvement, genetic recombination is a vital tool in creating new pre-breeding germplasm that can be used for improving existing cultivars, especially for complex traits such as stress tolerance. Lastly, while the analysis presented here is comprehensive, it is also limited by the availability of annotations for the metabolites identified. Majority of the metabolites with significant abundance differences (754 out of 1274 Pokkali-inherited metabolites, 32 out of 53 IR29-inherited metabolites, and 31 out of 50 unique FL510 metabolites) are unannotated. Such issues have been noted for shotgun metabolomics, and extensive testing and structural identification are required to properly identify these metabolites (Nakabayashi and Saito 2013; Schrimpe-Rutledge et al. 2016). Further scrutiny of these unknown metabolites may offer an opportunity to discover new biomolecules of importance for stress tolerance.

## Supplementary Information

Below is the link to the electronic supplementary material.

Supplementary file1 (DOCX 14 KB)

Supplementary file2 (PPTX 483 KB)

Supplementary file3 (PPTX 143 KB)

Supplementary file4 (PPTX 288 KB)

Supplementary file5 (PPTX 346 KB)

Supplementary file6 (XLSX 6048 KB)

## Data Availability

Data and/or materials used in the study are available from the corresponding authors on reasonable request. The metabolome and lipidome data will be available at the Dryad repository (10.5061/dryad.3ffbg79x5). The transcriptome data are deposited with links to BioProject accession number PRJNA1265305 in the NCBI BioProject database.

## Abbreviations

GA: Gibberellin
JA: Jasmonic acid
PCA: Principal component analysis
PLS-DA: Partial least squares-Discriminant analysis
TMM: Trimmed mean of M values
