# Supplementary material for: Constitutive metabolomic profile of a transgressive segregant of rice with superior salinity tolerance potentials due to unique morphological features and well-modulated growth
Source: Planta. 2025 Aug 29;262(4):92. doi: 10.1007/s00425-025-04811-0 (PMC12396997; doi:10.1007/s00425-025-04811-0)
Supplement: Supplementary file 1 — Supplementary file1 (DOCX 14 KB) [file 425_2025_4811_MOESM1_ESM.docx]

**List of Supplementary Materials legends**

**Suppl. Table S1** List of metabolites with significant induction or repression across the metabolome and lipidome datasets.

**Suppl. Fig. S1** Representative chromatograms of the rice genotypes in the different datasets generated in the study. Average chromatograms for each dataset were generated and compared for assessing the quality of the analysis. A combined sample (labeled QC_1) from the three genotypes (IR29, Pokkali, and FL510) were used as a primary quality control sample. This sample showed an average peak size for each dataset with respect to the other three genotypes, indicating that the data quality was sufficient for further analysis.

**Suppl. Fig. S2** Heatmaps of metabolites with significant induction or repression among FL510, IR29, and Pokkali. ANOVA with Tukey’s HSD post-hoc test was used to determine the metabolites that had significant repression or induction in the different genotypes (*P* < 0.05). **A** and **C** Heatmaps representing the positive and negative ion modes for the metabolome dataset. **B** and **D** Heatmaps representing the positive and negative ion modes for the lipidome dataset, with each column being the average log_2_-fold change from IR29. Each row is normalized and scaled. The heatmaps are reflective of the similarities and differences between the genotypes as seen in the PCA and PLS-DA plots in Fig. 1

**Suppl. Fig. S3** Expression of fatty acid biosynthesis genes in FL510, Pokkali, and IR29. These genes are involved in the initiation and extension of fatty acid chains. Expressions are shown in TMM along with pairwise comparisons for each genotype. Asterisks (*) denote significant differences (**, *P* < 0.01; *, *P* < 0.05).

**Suppl. Fig. S4** Expression of *TIFY* genes in FL510, Pokkali, and IR29. These genes represent the *TIFY* transcription factor family, which are directly related to jasmonic acid (JA) signaling. Expressions are shown in TMM along with pairwise comparisons for each genotype. Asterisks (*) denote significant differences (**, *P* < 0.01; *, *P* < 0.05).
