# Supplementary figures and images for: Constitutive metabolomic profile of a transgressive segregant of rice with superior salinity tolerance potentials due to unique morphological features and well-modulated growth
Source: Planta. 2025 Aug 29;262(4):92. doi: 10.1007/s00425-025-04811-0 (PMC12396997; doi:10.1007/s00425-025-04811-0)

## Slide 1
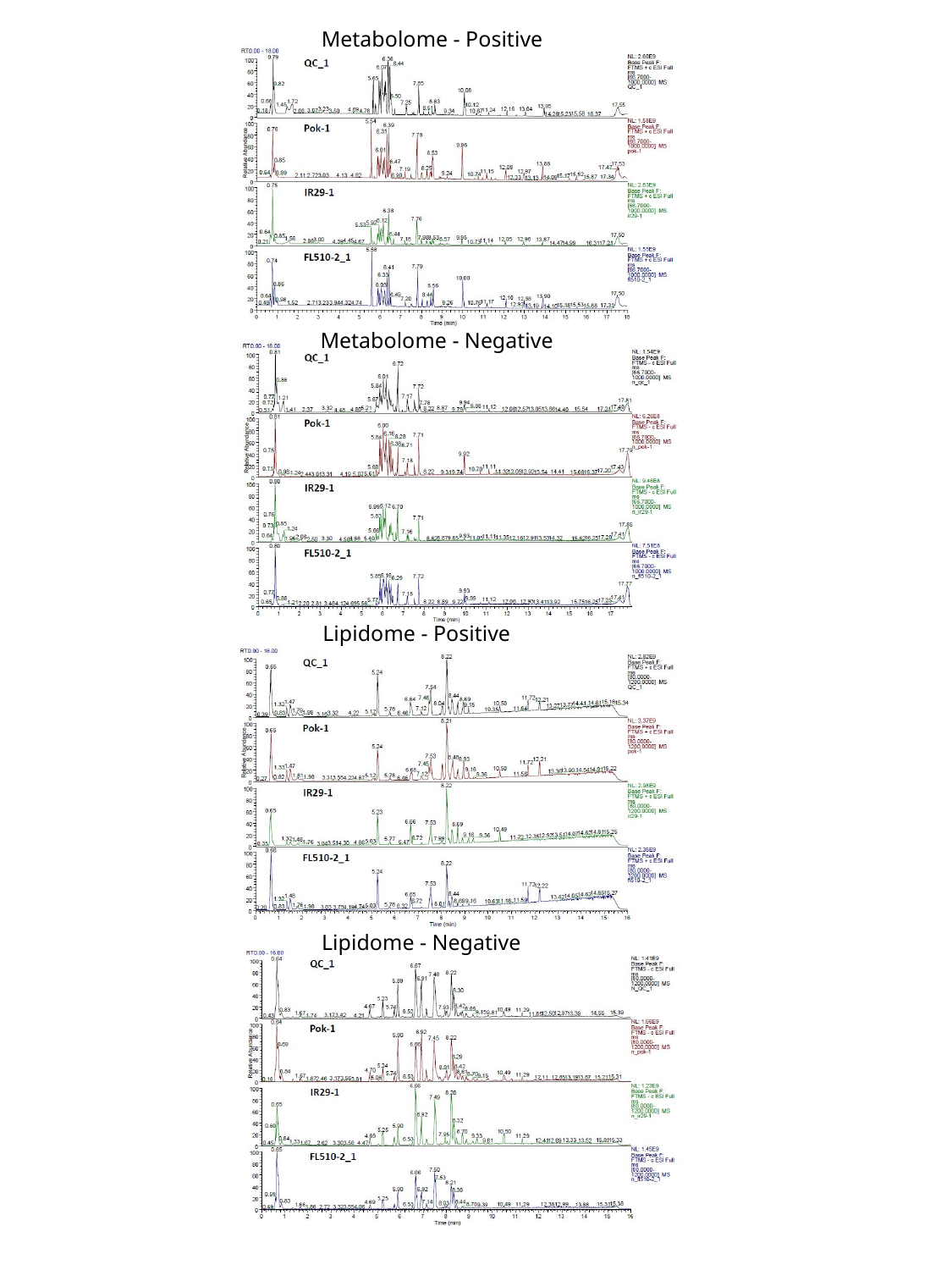

Metabolome - Positive
Metabolome - Negative
Lipidome - Positive
Lipidome - Negative

Supplement: Supplementary file 2 — Supplementary file2 (PPTX 483 KB) [file 425_2025_4811_MOESM2_ESM.pptx]

## Slide 1
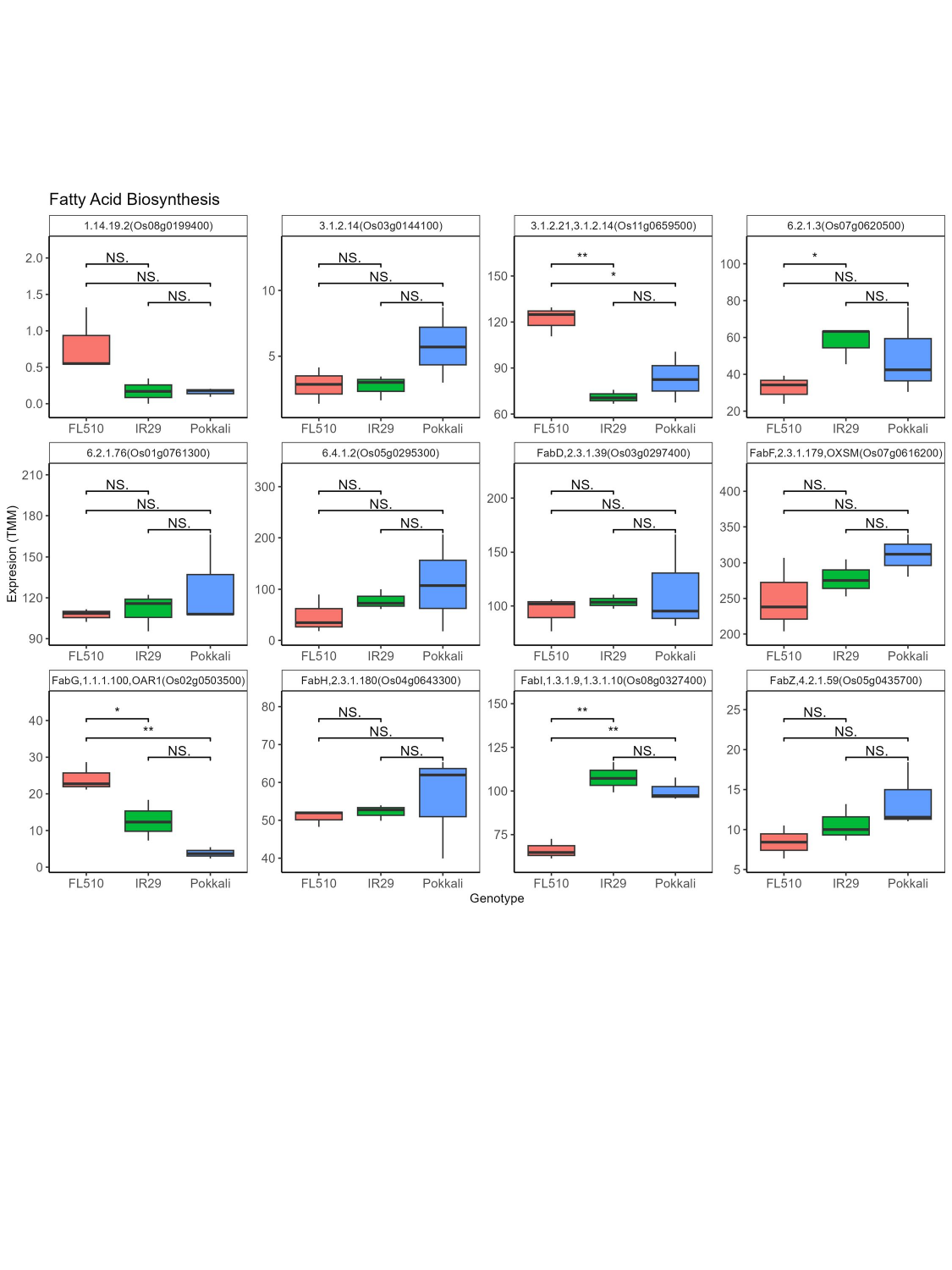

Supplement: Supplementary file 4 — Supplementary file4 (PPTX 288 KB) [file 425_2025_4811_MOESM4_ESM.pptx]

## Slide 1
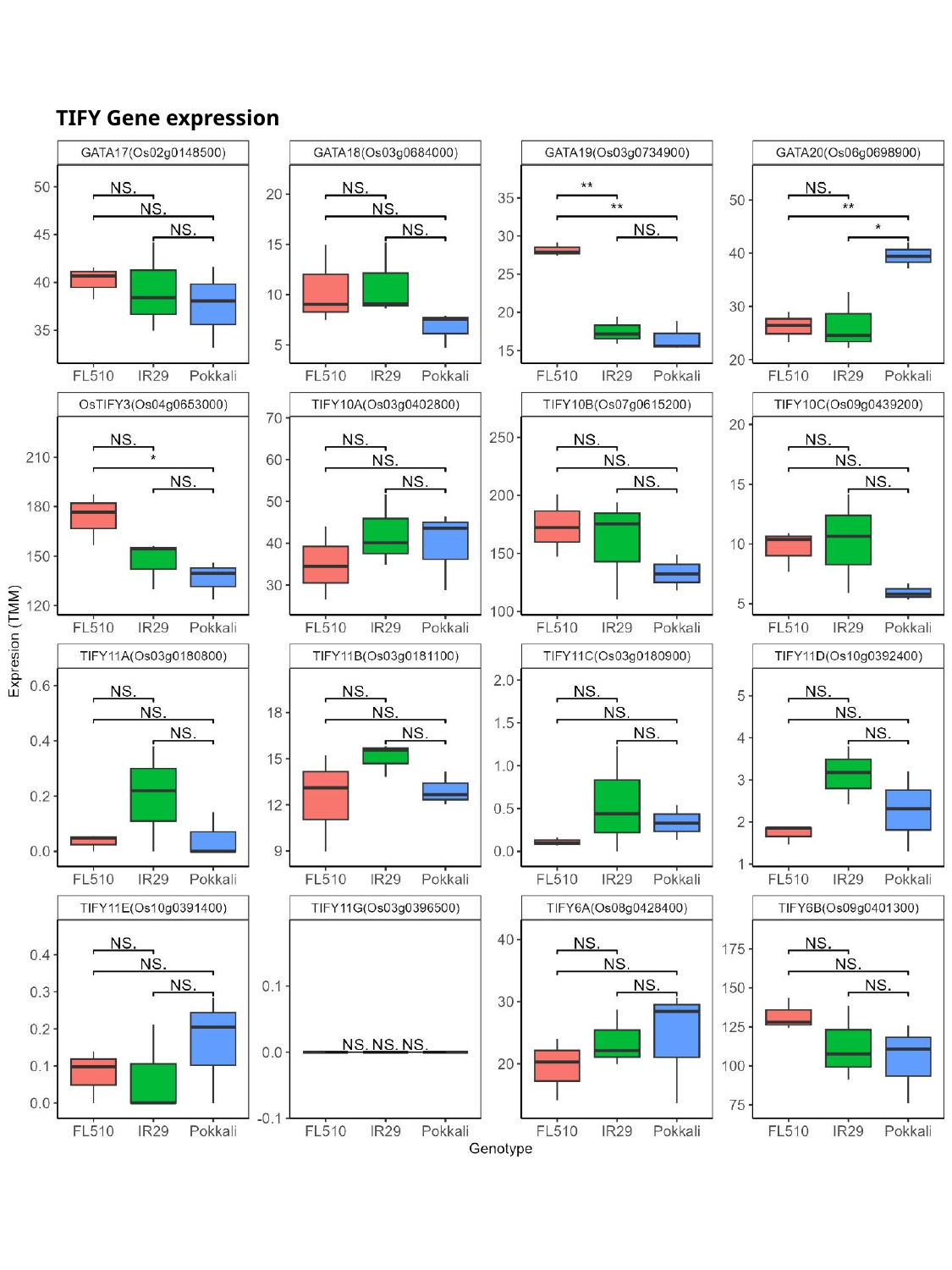

TIFY Gene expression

Supplement: Supplementary file 5 — Supplementary file5 (PPTX 346 KB) [file 425_2025_4811_MOESM5_ESM.pptx]
