# Supplementary material for: Constitutive metabolomic profile of a transgressive segregant of rice with superior salinity tolerance potentials due to unique morphological features and well-modulated growth
Source: Planta. 2025 Aug 29;262(4):92. doi: 10.1007/s00425-025-04811-0 (PMC12396997; doi:10.1007/s00425-025-04811-0)
Supplement: Supplementary file 3 — Supplementary file3 (PPTX 143 KB) [file 425_2025_4811_MOESM3_ESM.pptx]

## Slide 1
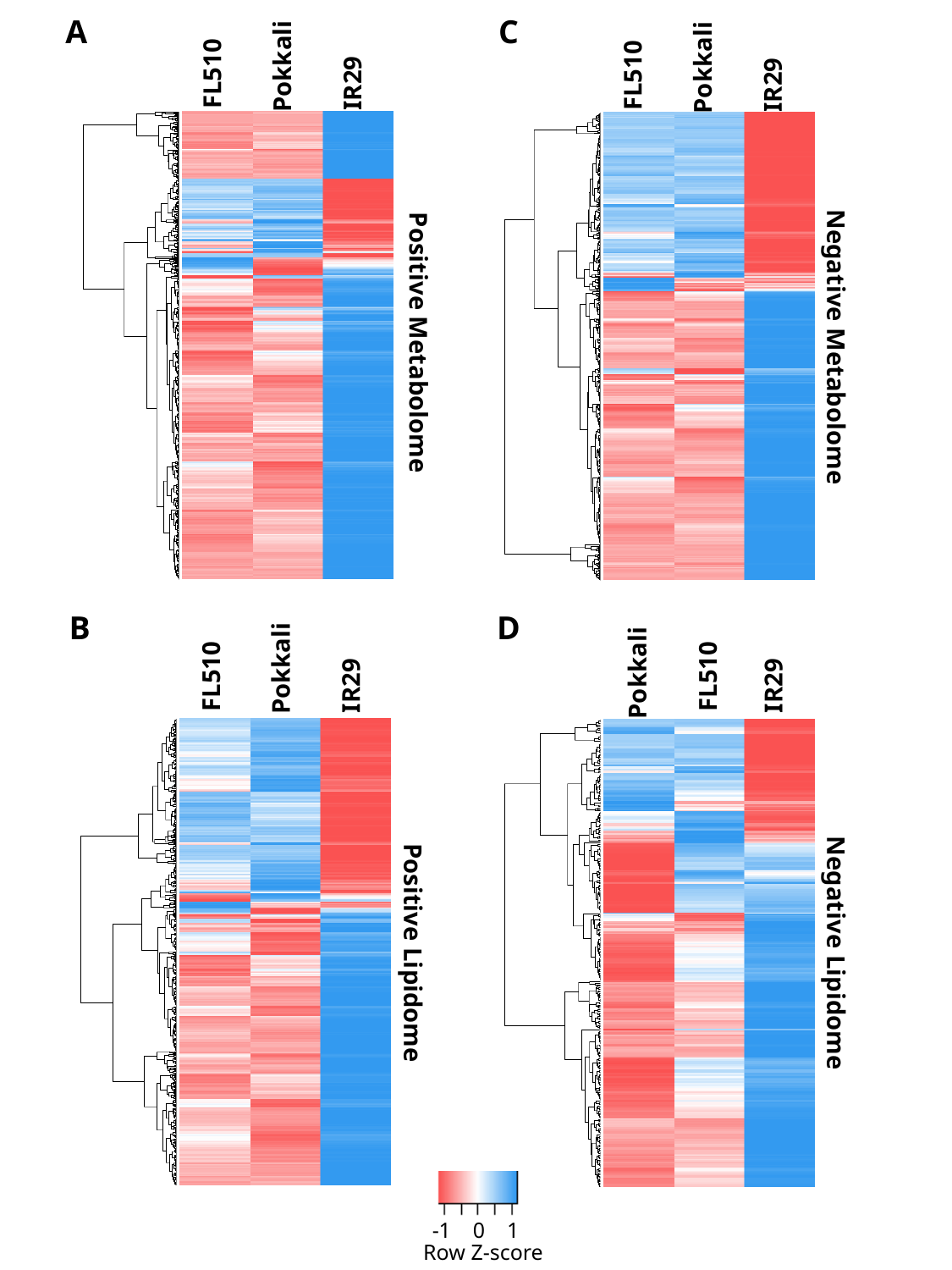

A
C
Pokkali
Pokkali
FL510
FL510
IR29
IR29
Positive Metabolome
Negative Metabolome
B
D
Pokkali
Pokkali
FL510
FL510
IR29
IR29
Positive Lipidome
Negative Lipidome
-1 0 1
Row Z-score
